# Elevational Distribution and Ecology of Small Mammals on Africa’s Highest Mountain

**DOI:** 10.1371/journal.pone.0109904

**Published:** 2014-11-05

**Authors:** William T. Stanley, Mary Anne Rogers, Philip M. Kihaule, Maiko J. Munissi

**Affiliations:** 1 The Field Museum of Natural History, Department of Science and Education, Chicago, Illinois, United States of America; 2 University of Dar es Salaam, Department of Zoology, Dar es Salaam, Tanzania; 3 Southern Highlands Conservation Programme, Mbeya, Tanzania; University of Colorado, United States of America

## Abstract

Mt Kilimanjaro is Africa’s highest mountain, and an icon for a country famous for its mammalian fauna. The distribution and abundance of small mammals on the mountain are poorly known. Here we document the distribution of shrews and rodents along an elevational gradient on the southeastern versant of Kilimanjaro. Five sites were sampled with elevational center points of 2000, 2500, 3000, 3500 and 4000 m, using a systematic methodology of standard traps and pitfall lines, to inventory the shrews and rodents of the slope. Sixteen species of mammal were recorded, including 6 shrew and 10 rodent species, and the greatest diversity of both was found at 3000 m, the elevational midpoint of the transect. No species previously unrecorded on Kilimanjaro were observed. Two genera of rodents that occur in nearby mountains (*Hylomyscus* and *Beamys*) were not recorded. *Myosorex zinki*, the only mammal endemic to Mt. Kilimanjaro, which previously was known by only a few specimens collected in the ericaceous or moorland habitat, was found in all but one (the lowest) of the sites sampled, and was one of the most widespread species of small mammal along the gradient. Two shrews (*Crocidura allex* and *Sylvisorex granti*) and one rodent (*Dendromus insignis*) were found throughout the entire transect, with *Dendromus* being observed at our highest trap point (4240 m). As in similar faunal surveys on other mountains of Tanzania, rainfall influenced the sample success of shrews, but not rodents. Trap success for rodents at 3500 m was notably low. This study contributes further justification for the conservation of the forest habitat of Mt. Kilimanjaro.

## Introduction

Knowing the distribution of organisms along an elevational gradient is critical to understanding the evolution and ecology of montane biotic systems, and to designing conservation strategies to maintain them. These reasons have motivated elevational surveys of small mammals in various areas of the world including Chile [1], Costa Rica [2], Malaysia [3], Philippines [4], [5], [6], Taiwan [7], and Tanzania [8]. Goodman, Ganzhorn & Rakotondravony [9] summarize some of the important biotic inventories along elevational gradients in Madagascar. Each of these studies elucidate both specific and broadly general patterns that help explain the mechanisms influencing the distributions of mammals along such gradients with significant implications for biogeographic analysis and conservation priorities [10]. Indeed, such surveys have served as vital baselines for comparison to subsequent inventories in testing the influence of climatic vicissitudes or habitat alteration. For example, range shifts in various mammalian species were documented in Yosemite Valley, California, with two similar surveys separated by almost a century [11].

Knowledge of the ecology and behavior of the targeted faunas help frame considerations of the results of systematic sampling along gradients. For example, Stanley & Hutterer [8] documented patterns of distribution along an altitudinal gradient in the Udzungwa Mountains of Tanzania that differed between shrews and rodents, and suggested that the amount of coincident rainfall influenced shrew, but not rodent, capture rates. Such observations must be factored into deciphering the results of systematic sampling along elevational gradients, and surveys using identical methodologies on other mountains should help to reveal whether such observations are unique to particular sites or more common across multiple gradients.

Mt. Kilimanjaro is the highest mountain in Africa and an icon for a region renowned for its unique mammalian fauna. Ironically, the mammals that inhabit the habitats of this volcano are relatively unknown, with most historical attention focused on larger species, leading to calls for complete inventories of the fauna of the mountain [12]. To date, the most comprehensive summary of our overall understanding of the mammalian fauna of Kilimanjaro remains that presented by Grimshaw, Cordeiro & Foley [13], who provided a faunal list of the mountain, and described past studies of Kilimanjaro’s mammalian fauna. Few studies employing systematic sampling have taken place on Kilimanjaro [14] and only one [15] used a systematic survey to document the presence and distribution of small rodents and shrews along elevational gradients on the mountain. The lack of detailed biotic vertebrate surveys, such as those of small mammals, hampers efforts to monitor ecological change over time on the mountain. Thompson *et al*. [16] suggest that climate change is affecting the habitat and ecology of Kilimanjaro, and baseline data for the distribution and abundance of various plants and animals are needed to judge the effect of such changes, as has been done elsewhere [11].

Using a standardized sampling regime that has been utilized in several other montane sites of Tanzania over the past two decades [8], [17], [18], [19] we surveyed the small mammals (shrews and rodents) at five different elevations and habitats along the southeastern versant of Mt. Kilimanjaro. Our study had three principal goals: 1) to initiate intensive surveys of the elevational distribution and abundance of small mammals along the transect sampled; 2) to test for differences between rodents and shrews in their relationship to elevation and response to different trapping methodologies; and 3) to compare the generated results to similar studies on Kilimanjaro and other mountains of Tanzania.

## Materials and Methods

### Study Site

**Figure 1:**
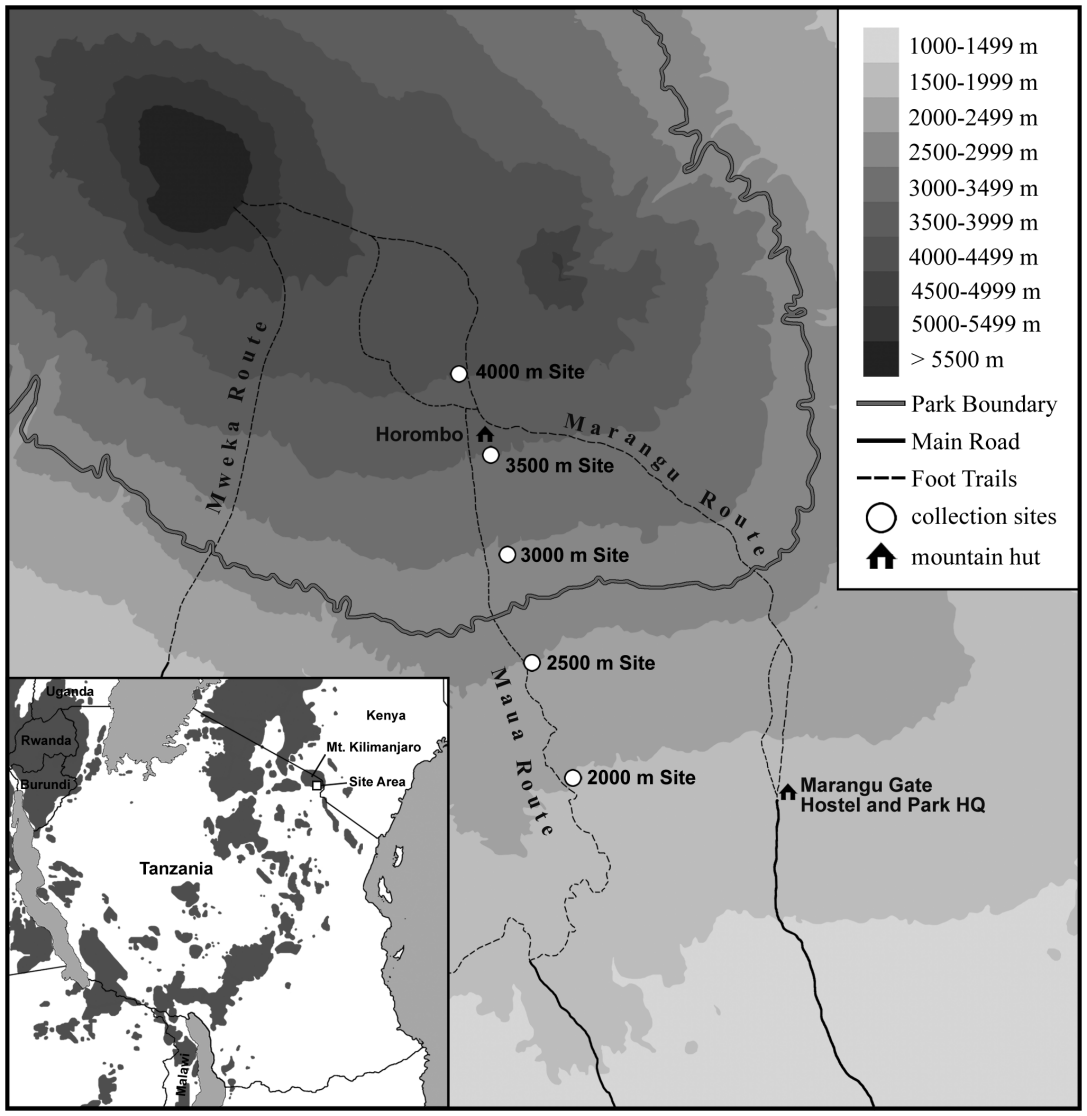
Map of Mt. Kilimanjaro showing routes, elevational contours and study sites.

Mt. Kilimanjaro is in northeastern Tanzania and reaches an elevation of 5895 m. An extinct volcano, the mountain is the conglomeration of three volcanoes: Kibo (the highest, most prominent and familiar), Mawenzi (the second peak of the mountain), and Shira (a plateau) [20]. Because the mountain is a popular destination for climbers, there are numerous paths that originate in the lowlands and run up the side of the mountain [21]. Two such routes that are on the southeastern (and wettest) versant are “Marangu” and “Mweka”. Between these two is the “Maua” path which is currently closed to tourists, and is used by Kilimanjaro National Park (KINAPA) staff to access and maintain facilities within the park. Between 17 July and 31 August 2002, we sampled the small mammals (shrews and rodents) at five different elevations, ranging from roughly 2000 to 4000 m, along the “Maua” route on the southeastern slope of Mt. Kilimanjaro (Figure 1).

The specific localities, elevations, habitats (sensu Mwasaga [22]) and dates of sampling are listed below. The elevations given for each site are centered at the associated camp and sampling efforts spanned roughly 100–200 m above and below the camp. For this reason, we labeled each camp at the closest 500 m interval (2043 = 2000 m; 2470 = 2500 m, etc.). Temperature and rainfall for each site (measured at camp) are listed in Table 1:

**Table 1:** Climatic data for each of the sites sampled on Mt. Kilimanjaro in July-August, 2002.

Elevation (m)	Daily Minimum Temperature (°C)	Daily Maximum Temperature (°C)	Daily rainfall (mm)
2000	8.5°±1.7	14.1°±2.1	4.2±3.0
	5–10°	12–18°	0–9
	N = 9	N = 8	N = 9 (8)
2500	3.6°±2.2	11.6°±1.9	1.6±0.3
	1–6.5°	9–15°	0–1.8
	N = 9	N = 9	N = 8 (2)
3000	2.2°±2.5	9.3°±2.5	1.5±3.1
	−2–5°	6–12°	0–9.5
	N = 9	N = 8	N = 9 (6)
3500	−0.9°±1.2	12.3°±3.5	0.1±0.1
	−3–1°	7.5–17°	0–0.2
	N = 9	N = 8	N = 8 (3)
4000	−6.8°±3.6	20.5°±5.9	1.5±3.4
	−12–−1°	11–25.5°	0–10.2
	N = 9	N = 8	N = 9 (3)

Totals given as mean ± standard deviation, range and sample size (number of days measured). Sample size for rainfall is given as number of days monitored and (number of days with rain).

Site 1–2000 m). 4 km N, 1.5 km W Maua, 3°14.404' S, 37°27.502' E, 2043 m; lower montane forest; 23–30 August 2002.

Site 2–2500 m). 7 km N, 2.5 km W Maua, 3°12.459' S, 37°26.818' E, 2470 m; upper montane forest; 17–25 July 2002.

Site 3–3000 m). 10.5 km N, 3.5 km W Maua, 3°10.627' S, 37°26.413' E, 2897 m; ecotone between montane forest and ericaceous zone; 26 July-03 August 2002.

Site 4–3500 m). 13.5 km N, 4 km W Maua, 3°08.941' S, 37°26.133' E, 3477 m; ericaceous zone; 4–12 August 2002.

Site 5–4000 m). 16 km N, 4.5 km W Maua, 3°07.566' S, 37°25.600' E, 3995 m; ecotone between ericaceous and alpine zones; 13–21 August 2002.

### Trapping Procedure

We used identical sampling techniques to those employed in similar small mammal surveys in other Tanzanian forests [8], [19], [23], [24], [25], [26], [27], [28]. Pitfall and trap lines were set in different microhabitats at each site, to sample shrews and small rodents (<200 g). Each pitfall line consisted of 11, 15 l buckets spaced 5 m apart, and placed so the upper rim was flush with the ground level. A 50 cm high vertical plastic fence was placed over the buckets, bisecting the openings. Most shrews and very small rodents were captured with this technique. Trap lines were installed using three types of traps: Museum Special traps, 14×7 cm; Victor rat traps, 17.5×8.5 cm (both manufactured by Woodstream Corporation, Lititz, Pennsylvania, USA); and medium-sized Sherman live traps, 23×9.5×8 cm (H.B. Sherman Traps Inc., Tallahassee, Florida, USA). Each line was composed of between 20 to 70 traps, with the Museum Special and Victor traps making up approximately 85% of each line. Traps were baited with pieces of freshly fried coconut coated in peanut butter, which was renewed each afternoon. Further details are outlined in Stanley, Goodman & Newmark [29].

All traps and buckets were checked once in the early morning and again in the late afternoon. Not all traps or buckets were employed for equal amounts of time (some trap lines were set the first day of the survey, others were installed on the second), so we use the measures “trap-night” and “bucket-night” (one trap or bucket in operation for one 24 hr period-0700 to 0700 hrs) to quantify sampling effort. We refer to the success rate of each method as either “trap success” or “bucket success”, and calculate these values by dividing the number of individuals captured by the number of trap-nights or bucket-nights and multiplying by 100. In discussions involving the two trapping methodologies combined, the term “sampling-night” refers to either one trap-night or one bucket-night, and “sample success” refers to the success rate for the two methodologies combined. The latter is calculated by dividing the number of individuals captured by the number of sampling-nights and multiplying by 100.

Standard external measurements and reproductive status were recorded for each specimen, which was then either prepared as a study skin and skeleton or preserved in 10% formalin, and later transferred to 70% EtOH. Specimens are deposited in the Field Museum of Natural History (FMNH) with a portion to be returned to Museum of Zoology, University of Dar es Salaam (UDSM). We follow the taxonomy of Carleton & Stanley [30], Holden [31], Hutterer [32], and Musser & Carleton [33].

### Ethics Statement

Permits for the collection and export of specimens were provided by the Tanzania Commission for Science and Technology (Ref# 2002-232-ER-90-172), the Tanzania Ministry of Natural Resources and Tourism (Wildlife Division; Ref# GD/R.40/1/22), and the Tanzania National Parks (Ref # TNP A44). Import of specimens into USA was approved by the US Fish and Wildlife Service (3177-W10214-9/18/02). Shrews and rodents were euthanized following the protocol approved by the American Society of Mammalogists [34], and the study was approved by the Field Museum of Natural History.

## Results

During the survey, we accumulated 11,562 sample-nights (8361 trap-nights and 3201 bucket-nights) and trapped 612 small mammals, including 319 shrews representing 6 species, and 293 rodents representing 10 species (Tables 2, 3, 4). Sampling success for shrews was significantly greater in buckets than in traps (*X* ^2^ = 695.2, P<0.05), and significantly more rodents were caught in traps than in buckets (*X* ^2^ = 44.8, P<0.05), a pattern observed in past studies on small mammals of Tanzania [8], [23], [24]. In 8361 trap-nights, 283 mammals were captured for an overall trap success of 3.4%. Of the mammals caught in traps, 263 were rodents (3.1% trap success for rodents) and 20 were shrews (0.2% trap success). In the 3201 bucket-nights, 329 mammals were captured for a total bucket success of 10.3%. Of these, 299 were shrews (9.3% success) and 30 were rodents (0.9% success). This striking pattern was evident not only across the entire survey, but also at each of the five sites sampled (Table 2). Shrew species caught in traps included *Crocidura allex*, *C. monax*, *C. olivieri*, and *Myosorex zinki* (weighing between 3.6–51.0 g). While most of the rodents caught in buckets were relatively small (i.e. *Dendromus insignis*; 7–20 g), both specimens of *Tachyoryctes daemon* (240–290 g) were captured in buckets. Other rodent species captured in buckets included *Grammomys dolichurus*, *Graphiurus murinus*, *Praomys taitae*, and *Rhabdomys dilectus*.

**Table 2:** Trapping totals for rodents and shrews by trap technique on the southeastern slope of Mt. Kilimanjaro in July-August, 2002.

Elevation	2000 m	2500 m	3000 m	3500 m	4000 m	Totals
**BUCKETS**						
# bucket-nights	616	649	649	638	649	3201
# individuals	84	75	86	51	33	329
(% bucket success)	(13.6)	(11.5)	(13.2)	(8.0)	(5.1)	(10.3)
# species	10	5	7	3	5	13
# shrews	68	74	79	48	30	299
(% bucket success)	(11.0)	(11.4)	(12.2)	(7.5)	(4.6)	(9.3)
# shrew species	5	4	4	2	3	6
# rodents	16	1	7	3	3	30
(% bucket success)	(2.6)	(0.1)	(1.1)	(0.5)	(0.5)	(0.9)
# rodent species	5	1	3	1	2	7
**TRAPS**						
# trap-nights	1600	1776	1785	1600	1600	8361
# individuals	67	68	57	3	88	283
(% trap success)	(4.2)	(3.8)	(3.2)	(0.2)	(5.5)	(3.4)
# species	5	9	11	2	4	12
# rodents	65	63	48	3	84	263
(% trap success)	(4.1)	(3.5)	(2.7)	(0.2)	(5.2)	(3.1)
# rodent species	4	7	8	2	3	8
# shrews	2	5	9	0	4	20
(% bucket success)	(0.1)	(0.3)	(0.5)		(0.2)	(0.2)
# shrew species	1	2	3	0	1	4
**TOTAL**						
# sample-nights	2216	2425	2434	2238	2249	11562
# individuals	151	143	143	54	121	612
(% sample success)	(6.8)	(5.9)	(5.9)	(2.4)	(5.4)	(5.3)
# species	11	13	14	4	6	16

**Table 3:** Elevational distribution of Soricomorpha species along the southeastern slope of Mt. Kilimanjaro in July-August, 2002.

Elevation	2000 m	2500 m	3000 m	3500 m	4000 m	Totals
Species						
*Crocidura allex*	24	19	40	45	30	158
*Crocidura hildegardeae*	7	0	0	0	0	7
*Crocidura monax*	21	29	26	0	0	76
*Crocidura olivieri*	2	2	0	0	0	4
*Myosorex zinki*	0	3	4	3	3	13
*Sylvisorex granti*	16	26	18	0^a^	1	61
Total # individuals	70	79	88	48	34	319
Total # species	5	5	4	2+1^a^	3	6
Total # sample-nights	2216	2425	2434	2238	2249	11562
Sample success (%)	3.1	3.2	3.6	2.1	1.5	2.7
Total # caught in buckets	68	74	79	48	30	299
Total # bucket-nights	616	649	649	638	649	3201
Bucket success (%) for pitfall lines	11.0	11.4	12.2	7.5	4.6	9.3

Only specimens caught in traps or buckets are included in totals.

a presence inferred from occurrence at lower and higher sites.

**Table 4:** Elevational distribution of rodent species along the southeastern slope of Mt. Kilimanjaro in July-August, 2002.

Elevation	2000 m	2500 m	3000 m	3500 m	4000 m	Totals
Species						
*Otomys angoniensis*	0	1	1	0	0	2
*Otomys tropicalis*	0	4	1	0^a^	7	12
*Dendromus insignis*	4	1	5	5	21	36
*Dendromus melanotis*	5	1	4	0	0	10
*Grammomys dolichurus*	3	6	6	0	0	15
*Lophuromys aquilus*	23	25	17	0	0	65
*Praomys taitae*	37	25	3	0	0	65
*Rhabdomys dilectus*	0	0	11	1	59	71
*Graphiurus murinus*	9	1	5	0	0	15
*Tachyoryctes daemon*	0	0	2	0	0	2
Total # individuals	81	64	55	6	87	293
Total # species	6	8	10	2+1^a^	3	10
Total # sample-nights	2216	2425	2434	2238	2249	11562
Sample success (%)	3.6	2.6	2.2	0.3	3.9	2.5
Total # caught in traps	65	64	48	3	84	264
Total # trap-nights	1600	1776	1785	1600	1600	8361
Trap success (%)	4.1	3.6	2.7	0.2	5.2	3.1

Only specimens caught in traps or buckets are included in totals.

a presence inferred from occurrence at lower and higher sites.

**Figure 2:**
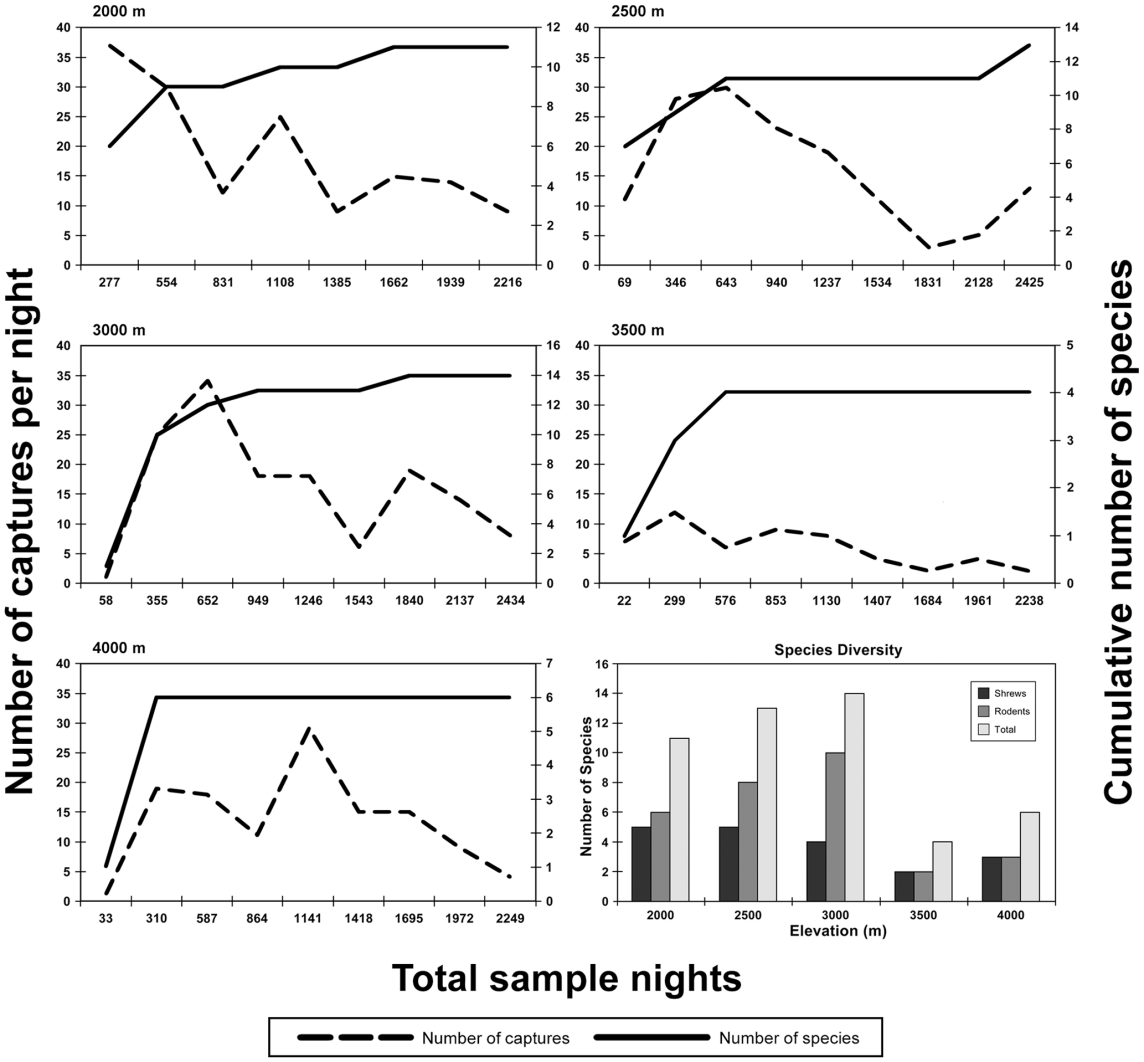
Species accumulation curves (for both pitfall and trap lines combined) for each site. The dashed lines represent the number of captures each day; the solid lines represent the cumulative number of new species for the site observed each day. The graph at the lower right shows the number of specimens of shrew, rodent and mammal captured at each site.

The number of captures (and overall sample success) at each elevational site ranged from 54 [2.4%] at 3500 m to 151 [6.8%] at 2000 m (Table 2). For shrews alone, the lowest values were observed at the 4000 m site (34 [1.5%]) and the highest values at the 3000 m site (88 [3.6%]; Tables 2, 3). For rodents, the lowest (6 [0.3%]) and highest (87 [3.9%]) values were observed at the 3500 m and 4000 m sites, respectively (Tables 2, 4). The cumulative number of species trapped reached an asymptote at all sites except 2500 m site (Figure 2), where *Dendromus insignis* and *Otomys angoniensis* were captured on the last day of trapping.

We examined the relationship of four daily capture parameters (number of individuals, number of species, number of new species [i.e. previously unsampled at a given site], and cumulative number of species) with cumulative sample-nights for both type of trapping methodology (Table 5) and mammalian order (Table 6). We chose cumulative sample-nights instead of day of sampling period because of the differences in sampling effort among sites (Table 2). Based on correlation analysis, there was a significant positive correlation between cumulative sample-nights and cumulative species across all sites for trap lines, bucket lines and both sampling methods combined (Table 5). The same pattern was generally evident at each site, although, in some cases, correlation values were high, but not significant. The correlation of the cumulative number of shrew species caught in buckets with cumulative number of bucket nights was significant at the 2500 m site, and high at all other sites. For both shrews and rodents, there was generally a negative correlation between cumulative sampling effort and new species captured. Notable exceptions include trap lines at the 3000 and 3500 m sites. There was no notable correlation between the number of species and cumulative sampling effort across the entire transect or at each site, with the exception of shrew species captured in buckets at the 2000 m site. The correlation between number of individuals and sampling effort varied among sites. There was a significantly negative pattern exhibited by pitfall lines and both trap and pitfall lines combined at the 3500 m site, but no such relationship exhibited at the 3000 and 4000 m sites. Table 6 presents the same analyses as Table 5, but is focused on the taxonomic groups sampled, and the patterns are similar.

**Table 5:** Product-moment correlation coefficients (*r*) of cumulative sample-nights with four parameters of trap/bucket captures.

Daily cumulative sample-nights correlated with (across)	Number of individuals	Number of Species	New Species added	Cumulative species
Total				
traps (rodents only)	0.099	−0.131	−0.428**	0.713**
traps (all captures)	0.068	−0.168	−0.491**	0.778**
buckets (shrews only)	(−0.249)	−0.085	−0.161	0.673**
buckets (all captures)	−0.149	0.168	(−0.217)	0.875**
traps and buckets combined (all captures)	−0.049	−0.100	−0.404**	0.798**
**2000 m**				
traps (rodents only)	(−0.698)	0.231	(−0.540)	0.865**
traps (all captures)	−0.746*	−0.066	(−0.605)	(0.577)
buckets (shrews only)	−0.800*	(−0.668)	(−0.684)	(0.577)
buckets (all captures)	−0.698	0.126	−0.882**	0.900**
traps and buckets combined (all captures)	−0.786*	−0.425	−0.752*	0.883**
**2500 m**				
traps (rodents only)	(−0.541)	−0.353	−0.376	0.727*
traps (all captures)	−0.455	−0.262	(−0.515)	0.776*
buckets (shrews only)	(−0.660)	−0.374	(−0.542)	1.000**
buckets (all captures)	(−0.657)	−0.429	(−0.611)	0.722*
traps and buckets combined (all captures)	(−0.604)	−0.442	(−0.591)	0.819**
**3000 m**				
traps (rodents only)	−0.424	−0.287	−0.452	0.809**
traps (all captures)	−0.342	−0.177	−0.401	0.826**
buckets (shrews only)	−0.020	0.164	(−0.585)	(0.548)
buckets (all captures)	0.060	−0.137	−0.666*	0.730*
traps and buckets combined (all captures)	−0.213	−0.220	(−0.539)	0.747*
**3500 m**				
traps (rodents only)	0.169	0.169	−0.126	0.907**
traps (all captures)	0.169	0.169	0.247	0.907**
buckets (shrews only)	0.809**	−0.365	−0.725*	(0.548)
buckets (all captures)	−0.760*	0.000	(−0.645)	(0.548)
traps and buckets combined (all captures)	−0.776*	−0.274	−0.754*	0.675*
**4000 m**				
traps (rodents only)	−0.310	−0.252	(−0.577)	1.000**
traps (all captures)	−0.309	−0.314	(−0.577)	1.000**
buckets (shrews only)	−0.465	−0.438	(−0.645)	(0.548)
buckets (all captures)	−0.439	−0.259	(−0.628)	0.903**
traps and buckets combined (all captures)	−0.108	−0.030	(−0.523)	(0.548)

Results are given for each sampling method for both targeted groups and everything captured. Values in parentheses represent strong but not significant correlations. *  = *P* ≤0.05; **  = *P* ≤0.01.

**Table 6:** Product-moment correlation coefficients (*r*) of shrew and rodent captures with four parameters of trap success.

Shrew and rodent captures correlated with (across)	Number of individuals	Number of Species	New species added	Cumulative species
Total, shrews	(−0.273)	−0.187	(−0.268)	0.726**
Total, rodents	0.074	−0.158	−0.496**	0.730**
2000 m, shrews	(−0.821)	(−0.668)	(−0.684)	(0.577)
2000 m, rodents	(−0.620)	0.063	0.724*	0.924**
2500 m, shrews	(−0.615)	−0.407	(−0.611)	0.722*
2500 m, rodents	(−0.539)	−0.412	−0.466	0.804**
3000 m, shrews	0.064	0.246	(−0.585)	(0.548)
3000 m, rodents	−0.440	−0.355	(−0.488)	0.794*
3500 m, shrews	−0.803**	−0.365	−0.725*	(0.548)
3500 m, rodents	0.452	0.000	(−0.518)	0.710*
4000 m, shrews	−0.398	−0.438	(−0.645)	(0.548)
4000 m, rodents	0.081	0.405	−0.411	(0.548)

Values in parentheses represent strong but not significant correlations. *  = *P* ≤0.05; **  = *P* ≤0.01.

**Figure 3:**
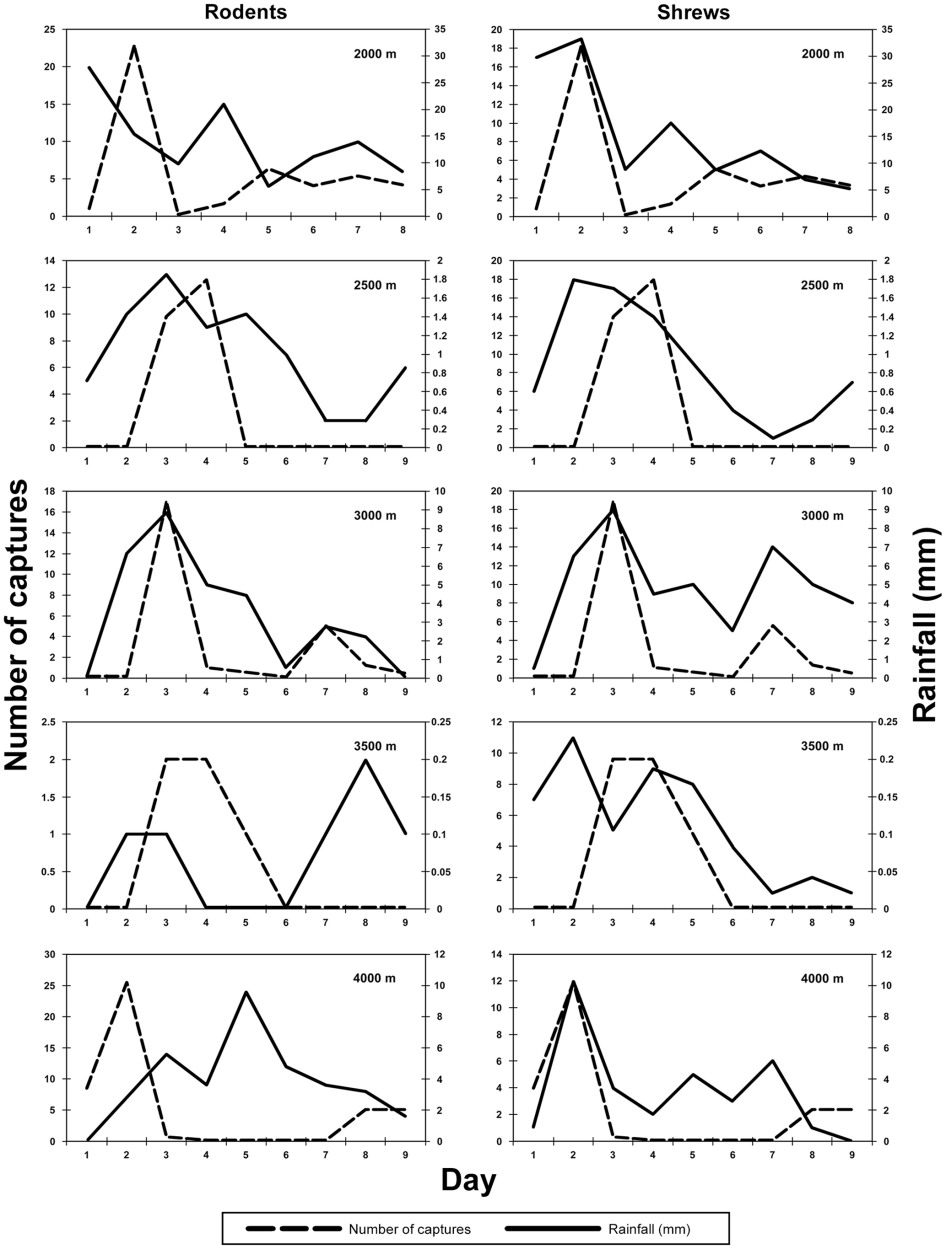
The relationship between numbers of individuals captured each day of the sampling period, and rainfall, at each site. Rodentia are on the left and Soricomorpha are on the right.

The effect of rainfall on captures is presented in Table 7. Generally, there was a stronger and more positive correlation between rainfall and daily captures of shrews, than there was for rodents. Over the entire transect, the capture of individual shrews in both buckets and traps was significantly correlated with the amount of rainfall each day, but the capture of individual rodents was not. A graphic representation of the differences between shrew and rodent captures with respect to rainfall amount is presented in Figure 3. The overall relationship between rainfall and captures of shrews was not as strong as in other elevational surveys of mammals in Tanzania [8].

**Table 7:** Product-moment correlation coefficients (*r*) of amount of daily rainfall with four parameters of shrew and rodent daily captures.

Rainfall amount correlated with (across)	Number of individuals	Number of Species	New species added	Cumulative species
Total, shrews (buckets and traps)	0.385*	0.422**	0.086	(0.277)
Total, rodents (buckets and traps)	0.190	(0.280)	(0.230)	0.053
2000 m, shrews	(0.523)	(0.655)	−0.025	0.262
2000 m, rodents	−0.108	(0.523)	0.424	−0.008
2500 m, shrews	(0.592)	(0.502)	−0.050	0.283
2500 m, rodents	(0.544)	0.201	−0.217	0.096
3000 m, shrews	0.719*	0.199	−0.246	0.187
3000 m, rodents	(0.644)	(0.487)	0.139	0.122
3500 m, shrews	0.363	0.378	−0.357	0.236
3500 m, rodents	−0.267	−0.060	0.286	0.334
4000 m, shrews	(0.629)	(0.652)	0.950**	−0.160
4000 m, rodents	−0.411	−0.086	0.927**	−0.160

All captures (both traps and pitfalls) of each group are included. *  = *P* ≤0.05; **  = *P* ≤0.01.

There was a significantly negative relationship between elevation and the total number of shrew species collected (Table 8). Additionally, elevation was negatively correlated with total number of individual shrews collected and sample success for shrews, with *r* values high, but not significant. Rodents showed no such notable pattern. The relationship between elevation and total sample success, number of individual mammals, and number of species collected for shrews and rodents combined was generally negative, but not significant. The least number of mammals, and species collected was at the 3500 m site. The greatest number of individuals noted was at the lowest site (2000 m), and the highest species diversity was observed at the 3000 m site. In most cases, the forested sites showed greater abundance and species diversity than the habitats above tree line (Tables 2, 3, 4).

**Table 8:** Product-moment correlation coefficients (*r*) between elevation and trap success.

Elevation correlated with	(*r*)	P
Total number of individual mammals collected	−0.59	>0.05
Total trap success	−0.59	>0.05
Total number of species collected	−0.68	>0.05
Total number of shrews collected	(−0.73)	>0.05
Shrew trap success	(−0.79)	>0.05
Total number of shrew species collected	**−0.95**	**<0.05**
Total number of rodents collected	−0.23	>0.05
Rodent trap success	−0.21	>0.05
Total number of rodent species collected	−0.56	>0.05

Values in parentheses represent strong but not significant correlations. Significant relationships (P<0.05) are in bold.

Captures in any individual trap or bucket were rare events. Although there was a 10.3% bucket success for all mammals captured, and 329 animals (299 shrews and 30 rodents) were collected in 385 buckets (77 buckets installed at each of five sites), most buckets captured no animals. Over the entire survey, 203 buckets caught nothing, 100 took one animal, 43 trapped two, 26 captured three, 7 caught four animals, 3 collected five animals, 2 trapped six animals, and ten animals were found in one bucket. Traps showed a similar pattern with 3.4% trap success in 1040 individual traps, and 283 captures (263 rodents and 20 shrews), but 834 traps caught nothing, 148 one, 42 two, 13 three and 3 four. To test for “trap competition” and to determine if captures were independent with respect to each other, we compared the observed distribution of captures by bucket and by trap to the Poisson distribution. Neither captures by buckets or traps followed the Poisson distribution (G-test for goodness of fit  = 84.0 for buckets, 10.0 for traps; p<0.01) suggesting a lack of trap or bucket independence. Significantly fewer traps or buckets caught one individual than would have been expected based on the assumption that the frequency of captures follows a Poisson distribution, and significantly more caught 2, or more, than expected [7].

## Discussion

**Figure 4:**
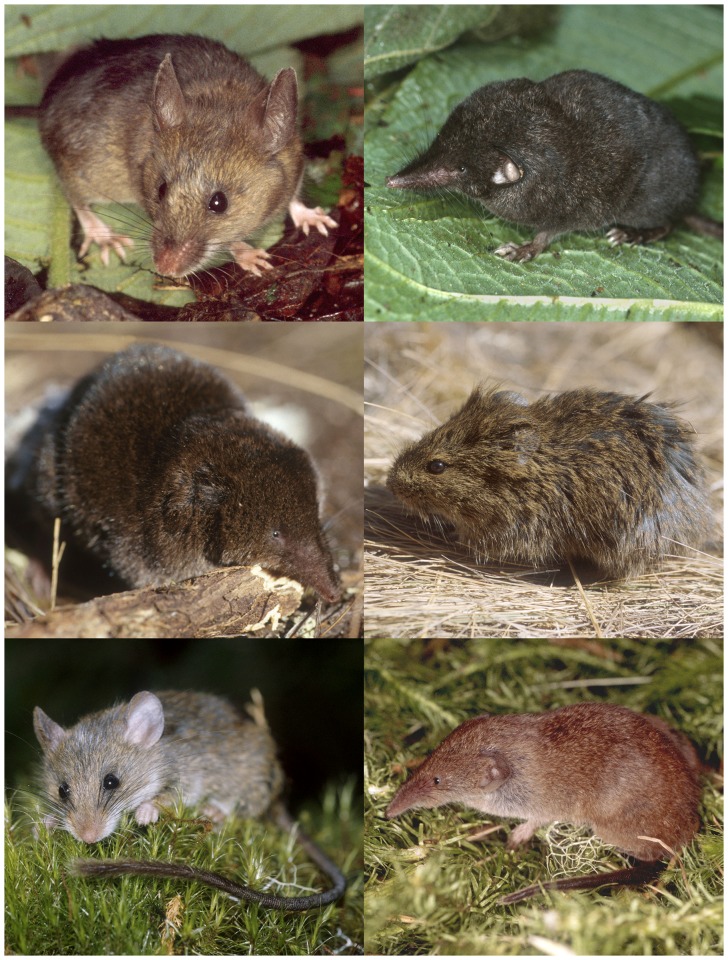
Six mammals found in the montane habitats of Mt. Kilimanjaro, Tanzania: *Praomys taitae* (top left); *Crocidura monax* (top right); *Myosorex zinki* (middle left); *Otomys tropicalis* (middle right); *Grammomys dolichurus* (bottom left); *Crocidura allex* (bottom right); all photographs by W. Stanley.

Sixteen species of mammal (6 shrews and 10 rodents) were recorded along an elevational transect from roughly 2000 to 4000 m on the southeastern slope of Mt. Kilimanjaro (images of select taxa are presented in Figure 4). Only one of these (*Myosorex zinki*) is endemic to the massif, and none were introduced taxa. The other species have broader distributions, to varying degrees. For example, among the soricomorphs, *Crocidura monax* has been recorded in neighboring mountains within the Eastern Arc Mountains to the southeast of Kilimanjaro, including the North Pare and West Usambara Mountains. *Crocidura allex* is known from other mountains of the northern highlands of Tanzania (Meru, Ngorongoro) and the highlands of Kenya (Kenya, Aberdares). *Crocidura hildegardeae* and *Sylvisorex granti* are distributed across Kenya and the montane habitats of the Albertine Rift. Finally, *Crocidura olivieri* is broadly distributed across much of the African continent [32]. Among the ten species of rodents recorded, most are variably distributed across eastern Africa, and some range over larger regions of Africa. For example, *Tachyoryctes daemon* is restricted to northern Tanzania, but murines such as *Grammomys dolichurus* and *Rhabdomys dilectus* range across much of eastern and southern Africa, as does the dormouse, *Graphiurus murinus* [31], [33]. However, many taxonomists have cautioned that some of these soricomorph and rodent taxa are almost certainly species complexes, and work in progress may alter our taxonomic understanding of these groups [31], [32], [33].

We found no species not previously documented on the mountain. There are several published faunal lists for Kilimanjaro, the most complete being that of Grimshaw *et al.* [13], who evaluated the accuracy of previous published records, and developed a working list of likely residents of the mountain. While this list includes every species we documented, there are other small mammals listed by Grimshaw *et al.* [13] that we did not document. For example, among shrews, we have no record of *Crocidura luna*. This species was listed by Grimshaw *et al.* [13] based on voucher specimens at the FMNH, collected at 1400 m, an elevation below our lowest sampling site (2000 m). Similarly, many rodent species listed by Grimshaw *et al.* [13] occur at elevations lower than the range of this study. Examples include genera such as *Aethomys*, *Arvicanthis*, *Lemniscomys*, *Mastomys*, *Pelomys* and *Tatera*. One rodent historically recorded in our elevation sampling range but absent from animals we captured is *Otomys typus* (* =  O. orestes zinki*; [35]). The holotype of *O. zinki* Bohmann 1943 was collected at Horombo Hut [36], [37], but the only two species of *Otomys* we documented were *O*. *angoniensis* and *O*. *tropicalis*. Two other rodents are notably absent from our inventory: *Beamys hindei* and *Hylomyscus arcimontensis*. Both are residents in forests of the Eastern Arc (including the North Pare Mountains roughly 50 km SE of Kilimanjaro) and Southern Highlands [38], [39], but no voucher specimen is known for either species from Kilimanjaro, or other northern highland sites. The type locality of *Beamys hindei* is Taveta, Kenya [40], and Dieterlen [41] identified a skull collected by C.G. Schillings in 1903 at Moshi as *Beamys*. Both localities are at, or near the base of Mt. Kilimanjaro. While this is not the most common species recorded in recent surveys of montane habitats of Tanzania across the elevational range from 600 to 2000 m [8], [19], given the number of trap nights expended during this survey, we anticipate the capture of *Beamys* if it occurs in the forests of southeastern Kilimanjaro. Records of *Hylomyscus* on Meru [42] and Ngorongoro [43] are now attributed to *Praomys taitae* [30], [38]. Recent surveys of both Meru and Ngorongoro using techniques identical to this study did not record either *Beamys* or *Hylomyscus* (Stanley, unpubl. data). All of this leads us to the conclusion that neither *Beamys* nor *Hylomyscus* currently occur in the forests of Mt. Kilimanjaro.

The trap success for rodents was highest at 4000 m and lowest at the 3500 m site (Table 4). The very low number of rodent captures at 3500 m (three in 1600 trap nights; Table 2) was striking, and is the lowest trap success recorded in similar surveys in montane habitats of Tanzania [8], [19], [24], [39]. Shore & Garbett [14] trapped at 3500 m, roughly the same elevation as our fourth site, but on the Shira Plateau on the western slopes of Kilimanjaro. The species they documented (*Crocidura allex*, *Myosorex blarina zinki* [ =  *M*. *zinki*], *Dendromus mesomelas kilimandjari* [ =  *D*. *insignis*] and *Rhabdomys pumilio diminutus* [ =  *R*. *dilectus*] were the same as in our study at the 3500 m site. One species (*Otomys tropicalis*) recorded by Shore & Garbett at 3500 m was not trapped by us at that elevation, but was collected at sites both lower and higher than 3500 m. Notably, their trap success for small mammals (2.7%; 81 captures in 2995 trap nights) was much higher that of the 3500 m site in this study (0.2%; 3 captures in 1600 trap nights), although the period of the surveys (mid-July to early August) was similar in both studies and trapping extended over several days at each site. The bait used by Shore & Garbett [14] included fried coconut and peanut butter (as in our procedure) but also fish, nuts and oats. However, the 3500 m site in our study was in stark contrast to lower and higher sites along the same transect, leading us to hypothesize that different bait is not the explanation for the lower rodent trap success at 3500 m in this study compared to the patterns documented by Shore & Garbett [14]. One potential explanation might be the proximity of Horombo Hut (3°8'20"S, 37°26'18"E) which was approximately 600 m from our trap lines (no other sites in this study were close to human habitation). The buildings and discarded flour and other foodstuffs generated by people occupying this touristic camp provide shelter and food for rodents. Indeed, while visiting Horombo on 8 and10 August, we saw many *Rhabdomys* moving between buildings. Both repeating our sampling methodology at our site, and sampling with the same techniques at, and at increasing distances from Horombo would be illustrative of the influence of human habitation on the abundance of native rodents in the environs of Kilimanjaro.

Mulungu *et al.* [15] published the results of two elevational transects of Kilimanjaro (along the Shira and Marangu routes) that focused on shrews and rodents. While total trapping effort was less than half of the current study (3600 vs 8361 trap nights), the recorded trap success was much higher (up to 36%). We attribute the higher success of Mulungu *et al.* [15] to the fact that traps were in place for only two nights at each site, and thus a reduction in capture rates typical of longer periods of time was not observed. The soricomorph and rodent species documented by Mulungu *et al.* [15] were identical to this study, with the exception of *Mus triton*, which they recorded at 2300 and 3270 m. *Lophuromys aquilus* was recorded at 3200 and 3590 m by Mulungu *et al.* [15] but our study did not record this rodent above 3000 m. However, one *Lophuromys* was brought to us by a Tanzanian National Parks employee who captured it at Horombo Hut (3760 m). The presence of other species at various elevations documented by Mulungu *et al.* [15] mirrored patterns observed during this study.

As in past studies within Tanzania, the combination of traps and pitfall lines were effective in sampling non-volant small mammal communities at different elevations on Mt. Kilimanjaro [8]. In general, species accumulation curves reached a plateau at each site, with the exception of the 2500 site where we captured *Dendromus insignis* and *Otomys angoniensis* for the first time during the last 24-hours of trapping. Notwithstanding the 2500 m pattern, we are confident that we documented almost all of the species of shrews and small rodents occurring at each site, and thus feel justified in comparing results among different elevational sites of this transect, as well as to results of similar surveys within Tanzania [8].

There was a significantly negative correlation between elevation and shrew species diversity at each site, and while not significant, there was generally lower abundance (as measured by sample success) for shrews as elevation increased (Table 8). However, rodents showed no notable correlation with elevation, either in diversity or abundance. This is in contrast to the patterns observed in the Udzungwa Mountains [8], where diversity and abundance of rodents were positively and significantly correlated with elevation. The same trends were not observed for shrews in the Udzungwas. Another difference between the Udzungwa and Kilimanjaro studies was seen in the overall measures of sample success in relation to elevation. Stanley & Hutterer [8] found either significant or high positive correlations between elevation and total sample success, number of individual mammals, and number of species collected for shrews and rodents combined. Such a relationship on Mt. Kilimanjaro was negative but not statistically significant. Whereas there was no mid-elevational peak (sensu McCain [10]) in the Udzungwa study, the greatest diversity of shrews and rodents on Kilimanjaro was at 3000 m, in the middle of our transect. Indeed, this site was situated at the ecotone between forest and heathland, and species typical of both habitats were present. For example, this was the highest, and lowest site where *Praomys taitae* and *Rhabdomys dilectus*, respectively, were documented and the only site where the two species were found together. In general, there was more species diversity for both shrews and rodents in forest habitats than above treeline (Tables 2,3,4).

Rainfall generally influenced the capture of shrews, but not rodents as was observed in the faunal inventories in the Udzungwa Mountains [8]. Thus, rainfall amounts while sampling shrew diversity or abundance should be considered. In addition, there was a lack of capture independence among traps and buckets across the entire transect and at each site. Stanley & Hutterer [8] documented similar results in the Udzungwa Mountains, and hypothesized that multiple captures are influenced by the placement of individual traps and buckets. More specifically, while traps cannot catch more than one animal, generally, buckets can capture more than one on a given bucket-night. The possibility exists that the presence of a captured animal in a bucket may attract other animals into that bucket.

The only endemic mammal, as currently understood, on Mt. Kilimanjaro is *Myosorex zinki* [44]. Until this survey, and that of Mulungu *et al*. [15], *M*. *zinki* was only known from a few specimens captured in the moorland habitats above tree line [14]. This species was documented across the elevational range of 2500 to 4000 m in this survey [45] and between 2500 to 2600 m by Mulungu *et al.* [15]. Thus, this endemic shrew extends across several different habitats on the mountain. *Myosorex zinki* was not observed at our lowest sampling site (2000 m). Stanley *et al.* [45] suggest that human impact on the forests at 2000 m on the Maua route may be responsible for the absence of this endemic mammal, but this hypothesis has not been adequately tested.

Three species (2 shrews and 1 rodent) were found at all sites sampled and occur across the range from roughly 2000 to 4000 m: *Crocidura allex*, *Sylvisorex granti*, and *Dendromus insignis*. The latter was found in the highest trap set in the survey (3° 6.481’ S, 37° 25.312’ E, 4240 m, on the ridge leading to West Lava Hill), and four individuals of this species were collected in this single trap (a Museum Special). How high small mammals extend on Kilimanjaro remains unanswered. Wild dog (*Lycaon pictus*) is the only mammal (other than *Homo sapiens*) recorded at the summit (5895 m; [46]). However no small mammal surveys have been conducted above 4000 m, and such efforts would help elucidate the upper ranges of shrews and rodents on this unique and iconic mountain, and would further our understanding of its faunistic dynamics.
